# Assessment of formal proceedings and out-of-court reorganisation: results from a survey among turnaround professionals in Austria

**DOI:** 10.1007/s10657-023-09771-y

**Published:** 2023-06-14

**Authors:** Stefan Mayr, Christine Duller, Matthias Baschinger

**Affiliations:** 1 Institute of Management Control and Consulting, Johannes Kepler University of Linz, Linz, Austria; 2 Institute of Applied Statistics, Johannes Kepler University of Linz, Linz, Austria

**Keywords:** Reorganisation, Turnaround professional, Bankruptcy law, Financial distress, Assessment, G 01, H 12

## Abstract

This study analyses the decision criteria for a specific form of reorganisation in a creditor-friendly bankruptcy system such as that of Austria. From a neoinstitutional perspective, we present different forms of bankruptcy law and the specifics of reorganisation in Austria. Next, we show several distinctive criteria and influencing factors for formal reorganisation and workouts. We group these factors into constitutions and institutional settings, process and handling, and implementation of the reorganisation. Using a sample of 411 survey responses from turnaround professionals, our empirical study analyses the decision criteria for a specific form of reorganisation. We apply a multivariate approach comprising two-sided paired samples Wilcoxon tests to assess the derived hypotheses and a hierarchical cluster analysis. Our results indicate significant differences in the valuation of the two forms: the turnaround professionals rate public perception much higher for out-of-court reorganisation, whereas legal certainty is rated significantly better for formal proceedings. Regarding process and handling, transparency and the handling of blocking positions are arguments for formal reorganisation, whereas flexibility is valuated better for workouts. In terms of implementation, respondents see advantages for out-of-court reorganisation, as it facilitates the implementation of both financial and operational measures. Taxation, the handling of blocking positions, and the improvement of public perception were identified as key development aspects for the legal framework conditions of the various reorganisation forms.

## Introduction

Bankruptcy and reorganisation represent a fruitful research field comprising many distinct strands (Staszkiewicz & Morawska, 2019). Such research has shown functioning insolvency law or reorganisation options for failing companies to be key success factors for countries. Both contribute, for example, to economic recovery after crises such as the 2008 financial and economic crisis or the recent COVID-19 pandemic (Stef & Bissieux, 2022). A reorganisation usually includes financial and operational measures (Sudarsanam & Lai, 2001) and aims at sustainably overcoming the crisis (Mayr et al., 2017). The literature has demonstrated differences in countries’ legislation (Blazy et al., 2008; Kaiser, 1996; Mruk et al., 2019), procedures (Blazy et al. 2020; Davydenko & Franks, 2008) and the use of formal reorganisation or out-of-court reorganisation (Blazy et al., 2014; Gilson, 1989, 1991; Kim, 2006). In recent decades, many European countries have not provided detailed legal regulations for out-of-court reorganisations or have done so only to a limited extent (European Commission, 2011a). To address this deficit, the European Commission has issued a directive to create a consistent framework for out-of-court reorganisation in the European Union (European Commission, 2019). The Commission has emphasised the importance of a preventive, out-of-court, restructuring framework applicable at the Union level. This framework should enable debtors to restructure in order to avoid insolvency and ensure the viability of their companies (European Commission, 2019). The Directive required Member States to adopt and publish the laws, regulations and administrative provisions necessary by July 17, 2021.

Failing companies must decide between formal proceedings and out-of-court reorganisation. Choosing a way to resolve default does not depend entirely on the companies' own willingness but happens under specific constraints, i.e., under the pressure of influential stakeholders (secured creditors such as banks, etc.). Previous studies have shown that this decision is very complex and multilayered (Blazy et al., 2014; Jacobs et al., 2012; Manzaneque et al., 2021). This decision relates to the type of corporation, the nature of distress, the firm's governance, or the managers' profile. In addition, the decision in favour of a particular form is also influenced by the turnaround professionals involved. On the one hand, they provide support in the form of legal or management expertise; on the other hand, they are also involved in negotiations with creditors.

The institutional setting in a specific country or legislation, e.g., efficiency of law response, costs of procedures or public perception of reorganisation, also impact this decision. Although the legal procedures involved in filing for bankruptcy vary considerably between countries, legislation is generally divided into two main groups: entrepreneur-friendly (also referred to as debtor-friendly) and creditor-friendly laws (Frouté, 2007). We focus on reorganisation in Austria, a country with a creditor-friendly bankruptcy system (Blazy et al., 2008). Substantial differences in the assessments of the reorganisation forms are to be expected for two reasons: for one, Austria has a pronounced reorganisation orientation in bankruptcy law (Konecny & Reisch, 2014); for another, it also belongs to the so-called prosecured creditor models as a more liquidation- and creditor-oriented system, as do Germany, Spain and Denmark.

Our review of the relevant literature indicates a dearth of research on the detailed and comprehensive decision criteria for a specific form of reorganisation. Previous studies comparing formal proceedings and out-of-court reorganisation methods mostly focus on isolated aspects, such as coordination problems and concentration of creditors (Blazy et al., 2014), the respective costs of proceedings (Gilson, 1991) or managerial incentives (Kim & Kwok, 2009). Other observed criteria include performance before default and the composition of debt (Yost, 2002). Since many details of both a legal and a business nature must be considered when deciding on a particular form of reorganisation, managers and entrepreneurs often call on the expertise of turnaround professionals (Decker, 2018; Kanter, 2003). The turnaround expert therefore contributes significantly to the decision-making process of the management/entrepreneur regarding the concrete design of the reorganisation and represent the link between the stakeholders and the distressed company.

Most studies use quantitative information that cannot capture opinions and assessments, especially considering the practitioners' point of view. The main contribution and originality of our paper is therefore to investigate how turnaround professionals assess the decision criteria for a specific form of reorganisation in a creditor-friendly bankruptcy system such as Austria. Deciding on a particular form of reorganisation is a complex process (Blazy et al., 2014; Manzaneque et al., 2021), with many different factors for decision-makers to consider. Thanks to their experience, turnaround professionals have a comprehensive understanding of the complexity of these decisions. They can also provide relevant insights into the underlying processes of reorganisation. To our knowledge, this is the first study to use qualitative data (perception by turnaround professionals) to analyse the decision criteria for a specific form of reorganisation. For this purpose, we first group the criteria and influencing factors used to distinguish different forms of reorganisation, i.e., distinctive criteria, into constitutions and institutional settings, process and handling, and implementation of reorganisation. The results of our empirical study cast light on the practical decision criteria for a specific form of reorganisation (which, in turn, are based on the distinctive criteria we developed) in a creditor-friendly bankruptcy system. The results further allow us to derive recommendations for policy makers and corporate practice.

A primary contribution of this paper is to cluster several distinctive criteria and influencing factors for a specific form of reorganisation. Furthermore, our results indicate significant differences in the assessment of the two forms: Turnaround professionals rate public perception much higher for out-of-court reorganisation, whereas legal certainty is rated significantly better for formal proceedings. Regarding process and handling, transparency and the handling of blocking positions are arguments for formal reorganisation, whereas flexibility is valuated better for workouts. In terms of implementation, respondents see advantages for out-of-court reorganisation, as it facilitates the implementation of both financial and operational measures.

The subsequent sections are structured as follows. Section 2 presents the theoretical background of the paper, including neoinstitutionalism, distinct forms of reorganisation, and details on types of bankruptcy law. Furthermore, we describe bankruptcy law and reorganization in Austria. Section 3 refers to distinctive criteria for a specific form of reorganisation (formal proceedings versus out-of-court) and develops the clusters researched in our empirical study. Section 4 describes the role and tasks of turnaround professionals in reorganisation, who represent the population from which we recruited the interviewees. Section 5 addresses the methodological aspects of our study, and Sect. 6 presents the empirical results. Finally, Sect. 7 discusses our results, and presents conclusions and recommendations for corporate practise.

## Theoretical background

### Neoinstituionalism

New Institutional Economy focuses on the analysis of institutions in which the exchange of goods and services takes place. The emergence of this approach arguably goes back to Coase (1937), who deals with the costs of a transaction and discusses them under the aspects of corporate growth and perfect markets. The concept of ‘the institution’ can be interpreted in different ways. In some cases, it is very broad and goes beyond the basic understanding of institutions as a demarcation from individuals. On the one hand, Commons (1950, p. 117) defines institutions as follows: "An institution is a going concern which engages in a series of transactions within the guidelines of a set of working rules. In this context, individuals can be both a part and a product of this going concern". This definition is thus based on the central aspects of the transaction, in the sense of the exchange of goods and services, and its rules as a set of guidelines for successful transactions. In addition to basic institutional rules, the behaviour of individuals is always influenced by the cultural context (Dugger, 1979). DiMaggio and Powell (1991), on the other hand, define ‘the institution’ as institutionalised elements of the formal structure of organisations and management practises that have an industry-wide, national or international spread.

North (1990), a representative of classical and neoclassical economics, describes institutions as "rules of the game in a society; the humanly devised constraints that shape human interaction". Accordingly, institutions comprise ‘formal institutions’ such as norms, laws and rules as well as ‘informal institutions’ like the conventions of a social group in general or of a company, which determine appropriate behaviour for the participants of the respective institution (Zucker, 1987). Formal institutions are easier and more quickly changed than informal ones (Williamson, 2000). Practical examples of formal institutions include property rights protection, tax rates and the availability of finance (Chowdhury et al., 2015). Informal rules, such as social norms and values, are embedded in human behaviour and often passed from one generation to the next (Chowdhury et al., 2015).

According to theory, changes in the formal structure of a company are therefore influenced less by competition or the quest for efficiency, but rather by the expectations and requirements of the business environment (DiMaggio & Powell, 1983). Consequently, neoinstitutionalist organisational theory rejects the model of an exclusively rational entrepreneur. Correspondingly, research interest thus focuses on the influence of institutionalised rules on organisations and their modes of action. These rules and other institutional aspects represent the independent variables in the analysis of an organisation, while the dependent variables comprise the design of the organisation and the response of managers to the institutions (DiMaggio & Powell, 1991).

In this context, Zucker (1987) emphasises that adapting to and complying with legal frameworks increases the probability of a company's survival. By complying with the law and norms, companies avoid legal disputes and sanctions, thus allowing them to concentrate on their core business. This also applies to financial crisis management and corporate reorganisation. Bankruptcy law and other relevant laws, e.g., company law, provide an institutional framework, influencing both the decision of an entrepreneur to initiate a reorganisation and the decision for a specific form of reorganisation (formal bankruptcy procedure versus out-of-court settlement). The institutional setting also impacts the behaviour of creditors and stakeholders during reorganisation as well as their willingness to continue to cooperate with a failing company and support reorganisation (Blazy et al., 2008; Franks & Torous, 1989). In addition to formal institutions, such as bankruptcy law, informal aspects will also influence the manager’s decision over a specific form of reorganisation: for example, negative public perception, stigmatisation in formal bankruptcy procedures, and the fear of image damage might impact this decision (Damaraju et al., 2020).

### Resolution of financial distress: formal reorganisation versus out-of-court settlement

Depending on the prevailing bankruptcy law framework, two distinct possibilities or procedural paths can be distinguished (Gilson, 1991). On the one hand, a formal reorganisation makes use of the bankruptcy law framework to resolve financial distress and restructure the company; on the other hand, an out-of-court settlement or a so-called "workout" or "out-of-court reorganisation"^1^ aims at remedying financial crises before the opening of judicial proceedings or outside bankruptcy proceedings. The latter path thus constitutes an alternative to the former.

Formal reorganisation is characterised by the principle of publicity and detailed legal provisions, which are regulated in the respective bankruptcy law proceedings. In this context, an international comparison shows that insolvency laws vary across the world (Blazy et al., 2008; Camacho-Miñano et al., 2013; Davydenko & Franks, 2008; Kaiser, 1996). Amongst other factors, these differences also seem to impact the decision for a specific form of reorganisation. Prior findings indicate that the institutional setting in a specific country or legislation affects the reorganisation strategy (Jacobs et al., 2012; Mutanen & Lehtimäki, 2009). Depending on the prevailing legal framework, formal proceedings can also be used strategically. They can be a mechanism through which firms make strategic changes that help to preserve value and overcome competitive disadvantages, such as unfavourable executory contracts (James, 2016).

In accordance with neoinstitutional theory, services provided by institutions incur costs. Thus, in financial distress, a firm’s decision to undergo formal reorganisation or out-of-court settlement would be determined by, among other factors, the corresponding costs, such as legal fees (Mruk et al., 2019). A distinction can be made between direct and indirect (bankruptcy) costs: Direct (bankruptcy) costs are all legal and administrative costs directly related to the proceedings (e.g., court costs, attorney fees) (Blazy & Stef, 2020; Mitter, 2015; Staszkiewicz & Morawska, 2019), while indirect costs include all opportunity costs arising from bankruptcy, such as deteriorated financing conditions of banks or adverse reputational effects (social costs) (Mruk et al., 2019; Sautner & Vladimirov, 2018). Case duration represents an important proxy of costs, as professional fees and costs rise with increasing case duration (LoPucki & Doherty, 2008). The value of the assets of the troubled firm, less all direct and indirect costs, is reflected in the recovery rates (Hotchkiss et al., 2008). The concept of costs is furthermore closely linked to judicial efficiency: Financial distress should ideally be resolved at the lowest possible cost, making the cost of bankruptcy is an important indicator of efficiency (Mitter, 2015). Judicial efficiency is determined by, among other factors, the demand for court services, which in turn is influenced by many other factors, such as the distribution of court costs among the parties involved (Voigt, 2016). In summary, the efficiency of a particular form of restructuring can be defined in terms of the value of the debtor's assets and the highest possible satisfaction of the creditors' claims, which is of course affected by the respective procedural costs.

Therefore, the decision between formal reorganisation and out-of-court settlement is influenced by the (expected) level of efficiency, with higher efficiency being preferred (Mruk et al., 2019). Since costs can be higher in formal proceedings than in workouts (Zafiris, 2018), this can lead creditors to avoid formal reorganisation, as they fear a poor outcome of the reorganisation (Mruk et al., 2019).

Another major disadvantage of court proceedings is the effect of the possible negative public perception and stigmatisation of the debtor resulting from bankruptcy (Damaraju et al., 2020). Stigma is defined as a mark of disgrace and discredit carried by a person (Singh et al., 2015) that arises from specific negatively connotated attributes (e.g., illness and disability) (Phelan et al., 2008) or from behaviours that violate social norms (Semadeni et al., 2008). Stigmatisation through bankruptcy is a social process in which individuals are held responsible for the failure of companies, which in turn negatively impacts their image and reputation (Wiesenfeld et al., 2008). Inasmuch as stigmatisation makes a restart more difficult (European Commission, 2011a; Lee et al., 2011), firms are likely to avoid stigmatisation, image damage and other social costs. In consequence, they often prefer to resolve financial distress in out-of-court settlements (Mruk et al., 2019). Out-of-court reorganisations are carried out discretely, without any formal publication, although distress and the planned reorganisation may be known to the public, for example, through media coverage (Cardon et al. 2011). Discretion makes it easier for the debtor to continue his or her business than in a formal insolvency procedure, where loss of goodwill tends to be very substantial (Garrido, 2012). Public perception might thus indirectly influence the decision between a formal reorganisation or an out-of-court settlement.

Detailed legal regulations for out-of-court reorganisations are available only to a limited extent or not at all. In Europe, according to an analysis of the European Union carried out in 2011, only 16 of 33 European countries investigated provided detailed legal regulations for out-of-court reorganisation (European Commission, 2011a). Such a lack of legal regulations can result in legal uncertainty in the implementation of reorganisation. To address this, the European Commission has issued a directive to create a consistent framework for out-of-court reorganisation in the European Union (European Commission, 2019). The Commission emphasises the importance of a preventive, out-of-court, restructuring framework applicable at the Union level. This framework should enable debtors to restructure to avoid insolvency and ensure the viability of their companies (European Commission, 2019).

The special preconditions of out-of-court reorganisations, such as discrete handling, may also explain the fact that significantly less research has focused on this form than on formal reorganisation. Prior research in that field has mainly focused on (US)listed companies (e.g., Gilson, 1989, 1991; Hotchkiss et al., 2008, 2014; Kim & Kwok, 2009; Yost, 2002).

### Different types of bankruptcy law

Although legal procedures involved in filing for bankruptcy vary considerably between countries, legislation is generally divided into two main groups: entrepreneur-friendly (also referred to as debtor-friendly) and creditor-friendly laws (Frouté, 2007). Businesses in general, and the results of insolvency processes specifically, can be significantly influenced by the pertinent legislation and by the protection given to debtors or creditors (Mruk et al., 2019). Studies show that bankruptcy laws that are lenient for entrepreneurs can positively affect the level of business activity in a country (Damaraju et al., 2020; Lee et al., 2007). Entrepreneur-friendly bankruptcy laws provide (formal) reorganisation procedures (Mruk et al., 2019), which offer a company some degree of legal protection while it is cancelling some of its debts and reorganising to compete more effectively (Peng et al., 2010). According to Peng et al. (2010) and Lee et al. (2007), entrepreneur-friendliness is characterised by the following criteria: the reorganisation option is available, an automatic stay on creditors´ claims during reorganisation is ensured, and entrepreneurs and managers are given the opportunity to remain on the job after filing for bankruptcy. In the event of liquidation, a fresh start is allowed.

In general, insolvency procedures in entrepreneur-friendly regimes are less time- and cost-intensive compared to creditor-friendly systems. Entrepreneurs and managers can retain their positions after filing for bankruptcy (Peng et al., 2010). This provision aims to prevent the premature liquidation of an insolvent company to protect its valuable (intangible) assets by preserving the management’s skills, experience and institutional knowledge (Zafiris, 2018). Some reorganisation systems apply the absolute priority rule—usually regarded as one of the creditor-friendly bankruptcy rules –, thereby encouraging the continuation of bankrupt companies without giving employees priority over company assets (Blazy et al., 2008). In this regard, Blazy et al. (2008) differentiate between social and entrepreneurial debtor-friendly models. By automatically suspending the claims of creditors, reorganisation systems favour the continuation of the company (Blazy et al., 2008). This limits the risks for failed entrepreneurs, and the exit and entry barriers are lowered (Lee & Yamakawa, 2012), thus increasing the probability that entrepreneurs will start again after bankruptcy (Peng et al., 2010).

The main features of creditor-friendly law are the preferential treatment of creditors over debtors in terms of securities (the company cannot sell the secured assets without the consent of the creditor) and priorities (normally the secured creditors are paid first) (Frouté, 2007). Liquidation is generally considered more creditor-friendly, as secured creditors are not prevented from asserting their securities, and the absolute priority rule applies in many regulations (prosecured creditor model and repressive model; Blazy et al., 2008). On the other hand, some regulations also allow deviations in absolute priority order within liquidative systems. Beyond protecting the rights of secured creditors, the debtor is usually not responsible for business operations during bankruptcy proceedings. However, some creditor-friendly bankruptcy laws allow staying in duty (Blazy et al., 2008). Blazy et al. (2008) distinguish between repressive and prosecured creditor models. It is argued that creditor-friendly systems impose higher indirect costs (Hotchkiss et al., 2008), which include both economic costs (in the form of financial loss) (Peng et al., 2010) and social costs (in the form of stigmatisation and image damage of the business) (Lee et al., 2007; Simmons et al., 2014). As a result, among other things, the levels of second-chance entrepreneurship are lowered (McCarthy et al., 2014), and the probability of failed entrepreneurs re-entering the market is reduced (Simmons et al., 2014). According to this framework, Austrian insolvency law can be classified as a prosecured creditor-oriented system. The following section describes its special features and recent changes.

### Bankruptcy law and reorganisation in Austria

Austrian bankruptcy law is traditionally counted among the creditor-oriented systems. According to Blazy et al. (2008), it belongs to the prosecured creditor model, which protects the interests of secured creditors. This classification does not include automatic stay on creditors’ claims and enforces the absolute priority rule. Since the functioning of Austrian bankruptcy law is comparably high and regulations are very detailed, the legal certainty of formal reorganisation in Austria can be regarded as high (European Commission, 2011b). Legal certainty encompasses a variety of aspects, such as contractual certainty or the reliability that obligations and legal norms will be both respected and enforced. In the context of bankruptcy (law), this means that managers can rely on contracts as well as on the certainty of compliance and efficiency of their enforcement by the courts in case of failure (Mruk et al., 2019).

Since its inception, bankruptcy law in Austria has undergone several reforms. In some cases, these have been mere adaptations, and in many cases, the reforms have also been based on concrete steering mechanisms for corporate practice, as was the case with the Bankruptcy Law Amendment Act (Insolvenzrechtsänderungsgesetz, 2010, hereinafter IRÄG 2010). The 1982 IRÄG, for example, introduced so-called "classless bankruptcy", which eliminated the distinction into creditor classes. Finally, the 1993 amendment explicitly regulated the bankruptcy of individual persons. The IRÄG 1994 made it easier to restructure within formal bankruptcy, as the deadlines for the fulfilment of quotas were doubled. The IRÄG 1997 represented a fundamental change in many areas: To reduce the number of bankruptcy rejections, it introduced relief for the payment of bankruptcy start-up costs. In addition, a review phase was introduced for corporate bankruptcies.

**Fig. 1:**
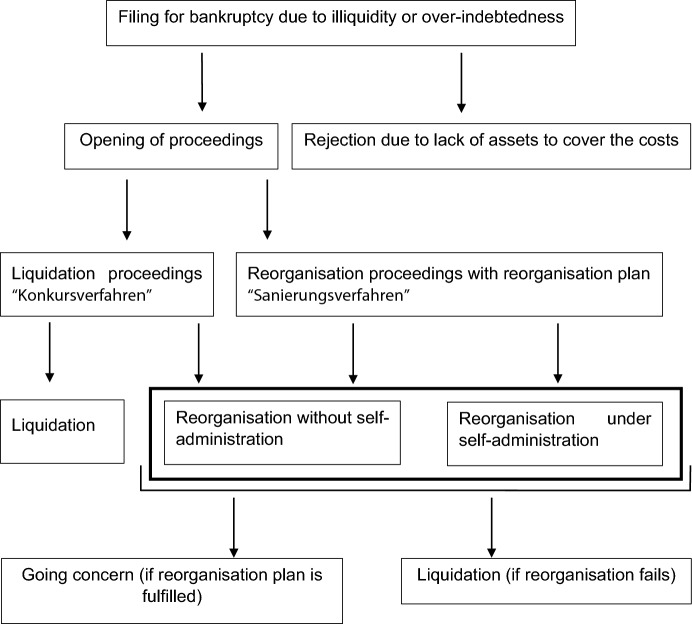
Overview over bankruptcy law and formal reorganisation for companies in Austria. *Source*: Our visualisation is based on formal reorganisation proceedings in Austria

The most significant change to the Insolvency Law Amendment Act (IRÄG) 2010, which came into force on July 1, 2010, was the abolition of the previous dual system consisting of the “Konkursordnung” (Bankruptcy Code) and the “Ausgleichsordnung” (Settlement Code). It retained the central characteristics of the Konkursordnung and individual provisions of the Ausgleichsordnung in the newly created “Insolvenzordnung” (Insolvency Code).^2^ The intention of the legislator was to facilitate the reorganisation of insolvent companies and thus create incentives for companies to file for bankruptcy earlier. The former reorganisation procedure, “Ausgleich”, which was hardly ever used at this point, and the “Zwangsausgleich”, which was relatively common in practice, were replaced by a newly designed and unitary reorganisation procedure (“Sanierungsverfahren”). The Insolvency Law Amendment Act 2017 (IRÄG) further facilitated debt relief for former entrepreneurs and private individuals by five simplifications: first, it shortened the duration of legal absorption to five years; second, it eliminated a minimum quota in the absorption procedure; and third, it eliminated the obligation to attempt to reach an out-of-court settlement to initiate proceedings even if the assets did not cover the costs. In addition, it increased the minimum remuneration of the bankruptcy administrator and adapted the regulation of international insolvencies to the European Insolvency Regulation. Figure 1 provides an overview of formal reorganisation in Austria to date.

The insolvency proceedings introduced by the IRÄG 2010 comprise two types of bankruptcy proceedings. On the one hand, it is possible to open liquidation proceedings (“Konkursverfahren”), in which the primary interest is the best possible satisfaction of creditors' claims with simultaneous liquidation of the company. However, a reorganisation plan can also be submitted during ongoing liquidation proceedings to carry out reorganisation. The liquidation proceedings are conducted by an administrator appointed by court.

On the other hand, it is possible to continue and restructure the company within the framework of reorganisation proceedings (“Sanierungsverfahren”). A distinction is made between whether the company remains in the hands of the entrepreneur (reorganisation proceedings under self-administration) or whether a court-appointed administrator takes over management of the company (reorganisation proceedings without self-administration). The central element of this type of reorganisation is the so-called reorganisation plan, which sets out how the required quota (minimum of 30% for self-administration, minimum of 20% without self-administration) can be met within two years. For private persons, debt relief is provided by using the debt settlement procedure (“Schuldenregulierungsverfahren”).

A petition for bankruptcy proceedings may be filed either by a creditor or the debtor. However, only the debtor can request the opening of reorganisation proceedings. In addition to illiquidity and/or over-indebtedness (in contrast to many other countries), the existence of cost-covering assets is a further prerequisite for the opening of proceedings. Cost-covering assets exist if the debtor's assets are sufficient to cover at least the start-up costs of the proceedings. If this is not the case, the proceedings will be rejected. In Austrian law, similar to other countries (Mruk et al., 2019), filing for bankruptcy is compulsory. If the company is insolvent (due to illiquidity and/or over-indebtedness), management must request a bankruptcy filing within 60 days of the date on which insolvency was known or should have been known. During the coronavirus crisis, the deadline for this obligation to file was increased to 120 days (according to the second Austrian COVID-19 law).

The essential principles of Austrian bankruptcy law include the equal treatment of creditors, a majority requirement for acceptance of the reorganisation plan by the creditors, and the discharge of (residual) debt of the debtor. Furthermore, there are publication obligations for the settlement of insolvency proceedings (Art. 74). Irrespective of whether the insolvency proceedings were opened as reorganisation proceedings or whether a reorganisation plan was submitted in the liquidation proceedings, the same requirements apply to the acceptance of this reorganisation proposal. For creditors who are entitled to vote, two further aspects are important: first of all, that they vote in support of the reorganisation plan (head majority); and secondly, that the total amount of outstanding claims of those voting in favour of the reorganisation plan exceed 50% of the amount of claims present (capital majority) (Art. 147). Regarding taxation, the so-called reorganisation privilege for reorganisation profits from formal proceedings applies (Art. 36 Austrian Income Tax Law), i.e. reorganisation profits must be taxed only on a quota basis.

Although the idea of reorganisation is deeply rooted in Austrian bankruptcy law, out-of-court reorganisation is also of great practical importance in Austria, with comparably high rates of success (Mayr et al., 2020; Mayr, 2018; European Commission, 2011b). In the study by Mayr, the analysis of 658 cases of out-of-court reorganisation shows a success rate of approximately 70%, calculated on the basis of the conclusion of a reorganisation agreement. The survey was based on the analysis of bank files, showing an average recovery rate for the banks of approximately 85%. The prevailing opinion is that out-of-court reorganisation in Austria is governed by the principles of equal treatment of creditors and unanimity. According to these principles, all creditors whose claims are not fully satisfied must agree to the settlement. With the implementation of the directive of the European Commission by the Austrian Restructuring Regulation (“Restrukturierungsordnung”, entry into force on July 17, 2021), the framework conditions and possibilities for out-of-court reorganisation have been significantly expanded. The central elements of the restructuring regulation are a restructuring officer appointed by the court, a restructuring plan, and the division into creditor classes. The new procedure is positioned between traditional court-supervised procedures and out-of-court reorganisation that were already being used. The extent to which this new procedure will be accepted in practice remains to be seen.

Since there is no legal obligation to engage turnaround professionals in Austrian reorganisations, both in and out of court, the fees for both restructuring forms in Austria are similar and essentially depend on the circumstances of the financial crisis (such as number of creditors and requirements for a restructuring concept). Regarding court costs, there is a substantial difference in the individual forms of reorganisation in Austria: In formal reorganisation, both the court costs and the costs of the administrator must be borne. In classic out-of-court reorganisation (without court), however, no court costs are incurred.

## Distinctive criteria and influencing factors for a certain form of reorganisation

The concrete decision for a particular form of reorganisation depends on several criteria and factors that have already been mentioned in Sects. 2.2 and 2.3., with 2.4 elaborating the specific situation in Austria. In addition, the literature mentions a multitude of advantages and disadvantages of the different forms of reorganisation (Blazy et al., 2008, 2014; Fischer & Wahrenburg, 2012; Gilson, 1989; Jacobs et al., 2012; Kim, 2006). In the following, we cluster the distinctive criteria identified in the literature, which we assume to influence the choice of the reorganisation form, into the following three groups: constitutions and institutional settings, processing and handling, and implementation. These criteria, however, vary across jurisdictions and cultural regions. The following argumentation is to be seen against the background of the Austrian prosecured insolvency law.

Constitutions and institutional settings (Jacobs et al., 2012; Mutanen & Lehtimäki, 2009), such as public perception, possible damage to the company’s image, legal uncertainty and taxation of reorganisation profits, strongly impact the behaviour of managers, creditors and other stakeholders in reorganisations (Blazy et al., 2008; Franks & Torous, 1989). Significantly, the public perception of bankruptcy, failure, and a specific form of reorganisation is a cultural issue (Singh et al., 2015). In countries with a negative public perception, negative reputational effects are created, such as a decline in demand or the repression and denial of the financial crisis by owners and/or managers (Mruk et al., 2019). This negative public perception will occur in the case of formal reorganisation due to publicity obligations in judicial proceedings. Moreover, in general, the public image of out-of-court reorganisations, even if they are publicly known, seems to be fundamentally better (Mayr, 2018; Staszkiewicz & Morawska, 2019). This leads to our first hypothesis:^3^

### H1

Turnaround professionals assess the public perception of out-of-court reorganisation significantly better than that of formal reorganisation.

Image damage that occurs due to the knowledge of insolvency among customers and suppliers in formal reorganisation can also lead to stigmatisation of the debtor (Damaraju et al., 2020). Even if cases of out-of-court reorganisation in large companies are well known to the public, the damage to the company's image is usually less than in formal reorganisations (Mayr, 2018). Individuals, such as managers and entrepreneurs, are held responsible for the failure. This makes it more difficult to restart or turnaround the company after reorganisation, if, for example, stigmatisation has made customers very sceptical about the company. Hence, we hypothesise the following:

### H2

Turnaround professionals assess the image damage for formal reorganisation significantly higher than for out-of-court reorganisation.

Legal certainty is related, on the one hand, to the efficiency of the implementation of bankruptcy law and, on the other hand, the extent to which legal regulations exist. Legal uncertainty resulting from shortcomings in the functioning of the judicial system (e.g., the absence of specific rules, an undefined order in which claims are to be met) may lead to a lower total recovery value and an aggravated holdout problem (Mitter, 2015), as well as to higher transaction costs (Chowdhury et al., 2015). The functioning of bankruptcy law in many countries, such as Austria, is high, and regulations are very detailed.

For out-of-court reorganisation, as has been mentioned, specific legal regulations do not exist in all countries. Additionally, Austria lacked concrete legal regulations until implanting the directive of the European Commission. Out-of-court reorganisations were characterised by the principle of equal treatment of creditors. Concrete regulations, mainly in insolvency law, primarily refer to the risks of these out-of-court reorganisations if negotiations fail. This leads us to the following hypothesis:

### H3

Turnaround professionals assess the legal certainty for formal reorganisation significantly higher than for out-of-court reorganisation.

Taxation in the context of reorganisation mainly refers to how reorganisation gains are taxed. In Austria, there is a so-called reorganisation privilege for reorganisation profits from formal proceedings. Reorganisation profits are taxed only on a quota basis, whereas profits in an out-of-court reorganisation must be fully taxed (Mayr, 2018). While beneficial tax treatment of reorganisation gains arising from debt relief can avoid lengthy and costly bankruptcy proceedings (Hotchkiss et al., 2008), tax disadvantages might have the opposite effect. Therefore, we hypothesise the following:

### H4

Turnaround professionals assess taxation for formal reorganisation significantly better than for out-of-court reorganisation.

The processing and handling of reorganisation are crucial to the success of reorganisation (Blazy et al., 2014). At the core is negotiation with creditors, especially with banks, since many companies, especially SMEs, strongly depend on and are predominantly funded through the assistance of banks (Mayr et al., 2020). In addition, the consent of all other stakeholders, in particular employees, suppliers and customers, must be obtained. Transparency in the process therefore increases the trust of all parties involved. Transparent and open communication encourages stakeholders, i.e., banks, to support distressed firms and to build trust and understanding (Mayr et al., 2020). In formal reorganisation, transparency is ensured by the insolvency judge or the insolvency administrator (European Commission, 2011a). On the other hand, flexibility in handling is of central importance to the process of reorganisation (Blazy et al., 2014). Flexibility means informal settlement with freely negotiable quotas and deadlines. Informal processing leads to a shorter period required compared to judicial proceedings (Mayr, 2018). Consequently, we derive the following hypotheses:

### H5

Turnaround professionals assess the transparency for formal reorganisation significantly higher than for out-of-court reorganisation.

### H6

Turnaround professionals assess flexibility for out-of-court reorganisation significantly higher than for formal reorganisation.

The alignment of different interests among creditors enables creditors to freely decide whether to participate in an out-of-court reorganisation of a firm or not. In contrast, in formal reorganisation, alignment of interests among creditors is accomplished and monitored by the court. The handling of blocking positions of single creditors represents a major challenge, both in formal reorganisation and out-of-court. As previous studies have shown (Fischer & Wahrenburg, 2012; Franks & Torous, 1989; Gertner & Scharfstein, 1991), the holdout problem is particularly prevalent in out-of-court reorganisations. This can make reorganisation, understood as a comprehensive agreement with creditors, difficult or impossible, prolong it, or lead to higher quotas for some creditors. This increases both the direct and indirect costs of reorganisation (Mruk et al., 2019; Staszkiewicz & Morawska, 2019). In accordance with these arguments, we propose the following:

### H7

Turnaround professionals assess the alignment of interest among creditors for formal reorganisation significantly better than for out-of-court reorganisation.

### H8

Turnaround professionals assess the problem of blocking positions for out-of-court reorganisation significantly higher than for formal reorganisation.

The third set of criteria comprises aspects of the implementation of the reorganisation (Laryea, 2010), especially with regard to the planned reorganisation measures. In implementation, the question arises as to what extent the necessary operational and financial measures can be implemented. Operational measures include cost reduction, sales generation, and operating-asset reduction. These measures generally form the first step of a turnaround strategy, whereas financial measures help firms reduce financial distress by executing asset divestments and improving cash flows. In this context, equity- and debt-based strategies can be distinguished. Hence, as previous studies have also shown, a mix of measures is usually implemented in successful reorganisations, both in and out of court (Blazy et al., 2014; Mayr et al., 2020; Sudarsanam & Lai, 2001). Since the forms of reorganisation differ fundamentally, this also affects the implementation of certain reorganisation measures. This results in the next hypotheses:

### H9

Turnaround professionals assess the possibility of implementing financial measures for out-of-court reorganisation significantly differently than for formal reorganisation.

### H10

Turnaround professionals assess the possibility of implementing operational measures for out-of-court reorganisation significantly differently than for formal reorganisation.

Since crises must be overcome in the long term, a key distinctive criterion rests on the assessment of whether sustainable reorganisation that includes elimination of the causes of the failure can be ensured. Sustainable reorganisation depends on whether the company has successfully overcome the underlying causes of the crisis and is able to fully engage in business again. Creditworthiness must be re-established, and the ability to meet financial obligations must be ensured. Over-indebtedness must be removed. With the help of strategic renewal, the restructured company must regain a competitive market position in the mid to long term (Mayr et al., 2017). In principle, sustainable reorganisation should be possible in both forms, but the ways to achieve this are certainly very different from a practical point of view. In out-of-court reorganisation, the restructuring concept and the reorganisation agreement with the main creditors determine long-term success. In contrast, in formal reorganisation, key decisions must be coordinated with the insolvency court or the insolvency administrator. This leads to an objective consideration of the circumstances but can also prevent strategic options. Hence, we hypothesise the following:

### H11

Turnaround professionals assess the possibility of ensuring sustainable reorganisation for out-of-court reorganisation significantly differently than for formal reorganisation.

**Fig. 2:**
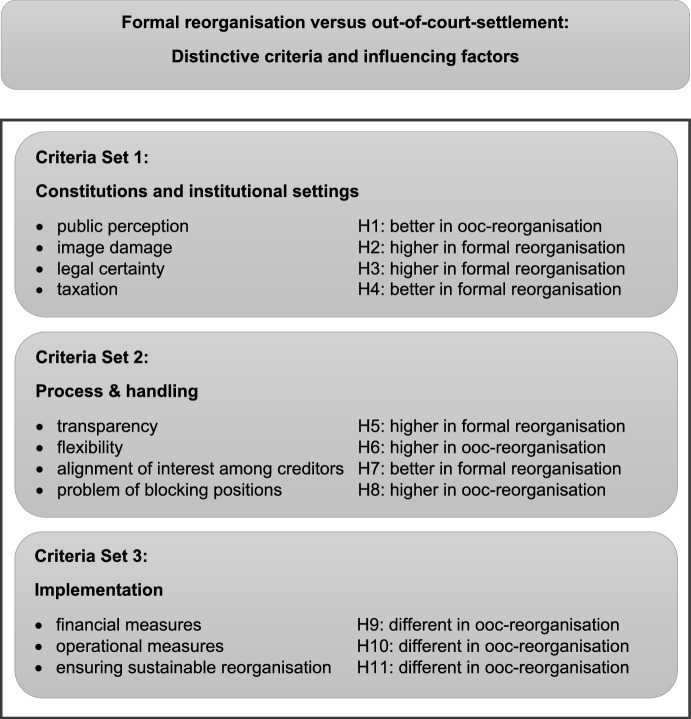
Distinctive criteria and influencing factors for a form of reorganisation. *Source*: Own elaboration

Figure 2 integrates and summarises the distinctive criteria for a form of reorganisation and summarises our hypotheses.

## Turnaround professionals in reorganisation

After presenting the assessments of turnaround professionals in the empirical part, we will first present the role of turnaround and professionals in reorganisation. Turnaround professionals are specialists in different fields of the turnaround or reorganisation process (Brownstein & Morris, 2002). They take the position of legal, management or business advisors (Decker, 2018) or act as interim managers of a distressed firm. In the latter case, the turnaround professional is included in the management of the firm in crisis to strengthen the creditability of the reorganisation procedure (Waisman & Lucas, 2008). In this case the turnaround expert contributes significantly to the decision-making process on the part of the entrepreneur/management regarding the concrete design of the reorganisation and represents the link between the stakeholders and the distressed company. The interaction between the firm and its stakeholders, as Bosse et al. (2009) suggest, should be based on fairness and trustworthiness. The task for a turnaround professional is to establish and maintain relationships with stakeholders and creditors to be perceived as a trustworthy partner (Bosse et al., 2009). It also falls to turnaround professionals to empower and inspire employees, who are an important group of stakeholders, in order to convince them that the initiated turnaround actions will be successful in the near future (Kanter, 2003). Equally, turnaround professionals are key to convincing creditors of the possibility of reorganisation. The chosen form of reorganisation and the detailed measures must be reasonable and appropriate from their individual point of view. In particular, creditors who take a blocking position and want to prevent reorganisation must be won over for support and considered in the planning of reorganisation (Mayr et al., 2020).

Additionally, decision-making processes are executed or supported by interim management or advisors. There are two ways of integrating turnaround professionals into the organisation: (1) As a board member (interim CEO such as CRO, Chief Restructuring Officer), the turnaround professional is responsible for navigating the distressed firm through reorganisation by leaving the remaining management free to concentrate on the operational business (Waisman & Lucas, 2008). (2) They can act as advisors to support management during the turnaround. In this case, the turnaround professional does not occupy a leading position but rather plays a consultative role. More specifically, turnaround professionals are experts in law, financial management and related topics. They possess comprehensive knowledge and experience, which they use to address different issues faced by distressed firms (Baird, 2014). According to a study by Nikolaou et al. (2007), specific knowledge in reorganisation and sufficient experience (up to 15 years) of turnaround professionals are key to a successful turnaround project (Nikolaou et al., 2007). As a consequence of these qualities, turnaround professionals significantly influence the choice between formal reorganisation and out-of-court settlement. Nevertheless, many other stakeholders, including managers, owners and shareholders, creditors or even the public, are also involved in the decision-making process and the choice of a certain form of reorganisation. Consequently, turnaround professionals are crucial, but they are not the sole decision-makers.

The main goal of a turnaround professional is to provide both short- and long-term solutions for financial problems in distressed firms (Brownstein & Morris, 2002). This can be achieved by identifying the company’s profitable core business and, subsequently, by improving the company’s financial statements (balance sheet, profit and loss, cash flow) with the help of financial and operational measures. Ultimately, securing and increasing the cash situation is essential to moving the distressed firm out of the crisis (Waisman & Lucas, 2008) and ensure a sustainable long-term reorganisation (Mayr et al., 2017). The concrete tasks and functions assumed by turnaround professionals strongly depend on the timing of hiring: If the firm is in severe financial trouble and on the brink of running out of cash, the opportunities for a turnaround professional will be limited, as the alternatives to bankruptcy will be very few (Brownstein & Morris, 2002; Conklin & Michelin, 2004).

In summary, one of the key tasks of a turnaround expert is to support management in deciding on a particular form of reorganisation (in court versus out-of-court). The advantages and disadvantages of formal reorganisation and out-of-court settlement are explained to the management and weighed against each other (for key decision-making criteria, see Fig. 2). Consequently, our empirical survey is based on turnaround professionals’ assessment of different forms of reorganisation.

## Data and methodology

### Sample: data collection and methodology

Our empirical study comprises an Austria-wide survey of turnaround professionals, conducted between January 2015 and June 2017. The study is based on a broad understanding of turnaround professionals, including management consultants, tax consultants, lawyers, bank representatives, entrepreneurs, investors specialising in restructuring, scientists, and representatives of the creditor protection association. The survey was conducted by means of questionnaires (see Appendix 1) in the form of a nonpersonalised online survey. In the first stage (January to February 2015), the questionnaire was sent to 700 addressees provided by the ReTurn network, which operates as an independent expert forum for restructuring, reorganisation and turnarounds in Austria. There was a return of 86 persons (12.3%), 73 of whom were ReTurn members. In the second stage, three subsequent surveys were conducted to gain further insights, especially into the assessment of non-ReTurn members. These subsequent surveys were performed in written or oral form with purposive samples. Of the total 411 respondents (online survey and subsequent surveys), approximately 19% are ReTurn members. As the representativeness of the entire sample cannot be guaranteed due to a lack of information on the total population and a relatively low response rate (possibly indicating a self-selection bias), all quantitative results should be regarded as exploratory but nonetheless meaningful. Using a low response rate to dismiss results as uninformative may be unwarranted (Meterko et al., 2015). Nevertheless, to verify the quality of the data, we controlled for non-response bias, comparing the first third of the respondents with the last third regarding profession, expert status and own firm size. There was no indication of non-response bias, as no significant differences between early and late respondents could be detected (Armstrong & Overton, 1977; Leslie, 1972).

The turnaround professionals were asked to give their assessment of the public perception, legal certainty and taxation of reorganisation, as well as the different aspects of implementation (financial or operational measures) connected with formal and out-of-court reorganisation. In addition, we obtained their assessment of practical handling regarding transparency for the affected stakeholders and flexibility of handling in the two forms of reorganisation. The surveyed aspects were chosen based on previously examined criteria for the assessment and delimitation of the two forms of reorganisation (see Sect. 3).

The questionnaire was pretested in advance and then slightly adapted. The assessment by the respondents was based on a five-point Likert scale, a method for measuring personal attitudes. The possible answers ranged from very negative to very positive, very low to very high, or very poor to very good, depending on the criterion. An uneven number of possible answers was deliberately chosen to allow a neutral assessment of specific criteria. Since the survey was conducted in Austria, the results must be assessed in the context of Austrian insolvency and company law (see Sect. 2.4).

For testing the (alternative) hypotheses H1 to H8 a one-sided paired samples Wilcoxon was applied, to test differences (H9 to H11) the two-sided paired samples Wilcoxon. In addition to Wilcoxon tests on central tendency for each criterion ordinal correlation coefficients Kendall-Tau-b and Spearman-Rho were calculated and tested to get information, if turnaround professionals who assess a special criterion positively for out-of-court reorganisation assess it negatively for formal reorganisation (discordant correlation) or positively as well (concordant correlation). For all tests the level of significance, i.e. probability of incorrectly rejecting the null hypothesis when it is actually true and confirming the alternative hypothesis, is □ = 5%. Finally, a hierarchical agglomerative clustering was used to form homogenous groups of criteria.

### Descriptive characteristics

Approximately 43% of the turnaround professionals surveyed work as management consultants, and approximately 42% work as tax consultants. Due to the possibility of multiple answers, there is an overlap in the areas of work. For example, 14% of the professionals are management consultants as well as tax consultants (56 cases). Some of the management consultants are also active as managers, some of them on executive bodies. Approximately 18% of those surveyed are lawyers, while approximately 8% are bank representatives specialising in restructuring. Approximately 12% of those surveyed are active as entrepreneurs (with overlaps to other tasks), while only 4% of those surveyed are investors who specialise in restructuring. The sample also includes scientists, representatives of creditor protection associations and financial service providers to a lesser extent (Table 1).

**Table 1:** Descriptive characteristics

Variable	Valid cases	Value	Cases	Percent
Profession	411	Management consultant	177	43
		Tax consultant	174	42
		Lawyer	72	18
		Entrepreneur	49	12
		Bank representative	34	8
		Investors in restructuring	18	4
Expert	409	No	301	74
		Yes (≥ 50% activity in reorganisation)	108	26
Clients’ size	406	Small (0–49 employees)	249	61
		Medium (50–259 employees)	109	27
		Large (≥ 250 employees)	48	12
Own firm size	410	Small (0–49 employees)	319	78
		Medium (50–259 employees)	35	9
		Large (≥ 250 employees)	56	14

The arithmetic mean and the median of the respondents’ restructuring experience are approximately 17 years. Many management consultants are also active in financing or transaction consulting. In the case of tax consultants, involvement with reorganisation often represents only a comparatively small part of their overall activity. The following values represent the share of corporate reorganisation in their overall activity: The average share of reorganisation in the overall activity is approximately 30%, whereas this share is significantly higher (more than 60%) among the members of the ReTurn network.

The company size of the clients of the surveyed experts—according to the definition of the European Commission (EU Commission 2013, notified under document number C(2003) 1422)—is distributed as follows: 61% of the clients’ companies are small enterprises (0–49 employees), 27% are medium-sized companies (50–249 employees) and 12% are large companies (250 or more employees). The fact that large enterprises are clearly overrepresented compared to the population indicates that a comparatively large number of “resources” is invested in the reorganisation of large enterprises. However, it also shows that restructuring advice is crucial for small and medium-sized enterprises.

## Results

In the following, the results of the empirical survey regarding turnaround professionals’ assessments are presented, visualised and interpreted in detail. We test our postulated (alternative) hypotheses using one-sided (H1 to H8) or two-sided (H9 to H11) paired samples Wilcoxon tests (with α = 0.05). The divided into the three subsections constitutions and institutional settings, process and handling, and implementation. The fourth and last subsection shows the results of the multivariate approach, i.e., a hierarchical cluster analysis.

### Constitutions and institutional settings

**Fig. 3:**
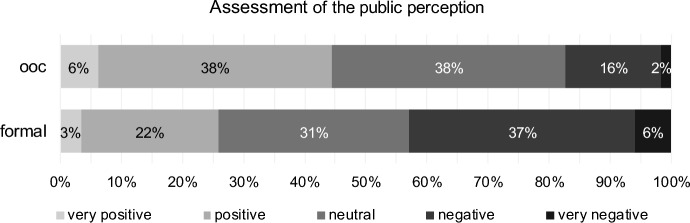
Assessment of public perception of out-of-court reorganisation (ooc) and formal reorganisation (formal)

Figure 3 shows the respondents’ assessments concerning public perception of formal reorganisation and out-of-court reorganisation. Almost half (44%) of the surveyed professionals rated the public perception of out-of-court reorganisation (ooc) as very positive or positive, and only 18% rated this form of reorganisation as negative or very negative. Concerning formal reorganisation (formal), the public perception is rated very positive or positive by approximately 25%, while more than four of ten (43%) assessed it as negative or very negative.

The results (see Table 2) indicate that public perception of out-of-court reorganisation is considered significantly better than that of formal reorganisation by turnaround professionals (paired Wilcoxon Test, *p* value one-sided = 0.000, H1 confirmed). This result, as well as the following, is to be seen against the background of the Austrian prosecured insolvency law, which, due to its characteristics, favours creditors rather than the entrepreneur.

**Table 2:** Results for public perception (H1) for out-of-court reorganisation (ooc) and formal reorganisation (formal)

Test	*p* value		Statistics (405 valid cases)		Decision
Paired Wilcoxon Test	One-sided	0.000	# ooc better	211	H1 confirmed
			# ooc equal formal	106	
			# ooc worse	88	
Correlation	Two-sided	0.046	Kendall-Tau-b	− 0.085	Negligible correlation
		0.054	Spearman-Rho	− 0.096	

Ordinal correlation coefficients indicate that turnaround professionals who assess public perception positively for out-of-court reorganisation assess it negatively for formal reorganisation (discordant correlation), significant using Kendall-Tau-b, not significantly measured with Spearman-Rho, but in both cases on a negligible level.

Tax consultants assessed public perception of out-of-court reorganisation worse than other professionals (Kendall-Tau-b = 0.185, *p* value < 0.001). Experts—which are defined having at least 50% of their activity in reorganisation—assessed it better than non-experts (Kendall-Tau-b = − 0.214, *p* value < 0.001). The bigger the clients’ size the better the assessment of public perception of out-of-court reorganisation (Kendall-Tau-b = − 0.248, *p* value < 0.001). The influence of client’ size is quite opposite for the assessment of public perception of formal reorganisation (Kendall-Tau-b = 0.104, *p* value = 0.02).

**Fig. 4:**
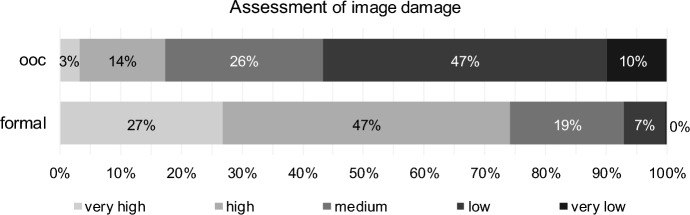
Image damage for out-of-court reorganisation (ooc) and formal reorganisation (formal)

According to the respondents, potential damage to the image of a firm as a result of formal reorganisation is considerably higher than that of out-of-court reorganisation. Approximately 74% estimate image damage during formal reorganisation as very high or high (Fig. 4), and only one turnaround professional assessed the image damage as “very low” in the case of formal reorganisation. This seems to confirm the general thesis of stigmatisation in insolvency. For out-of-court reorganisation, only 17% of respondents perceive high or very high damage to the image of a firm.

Image damage of formal reorganisation is considered significantly higher than that of out-of-court reorganisation by the turnaround professionals (paired Wilcoxon Test, *p* value one-sided = 0.000, H2 confirmed, see Table 3). Correlations between the assessments are significant but (again) negligible.

**Table 3:** Results for image damage (H2) for out-of-court reorganisation (ooc) and formal reorganisation (formal)

Test	*p* value		Statistics (404 valid cases)		Decision
Paired Wilcoxon Test	One-sided	0.000	# formal higher	310	H2 confirmed
			# ooc equal formal	73	
			# formal lower	21	
Correlation	Two-sided	0.033	Kendall-Tau-b	0.092	Negligible correlation
		0.029	Spearman-Rho	0.109	

**Fig. 5:**
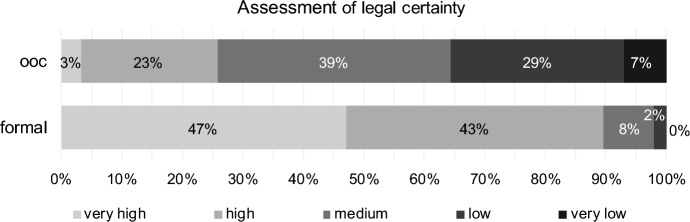
Legal certainty for out-of-court reorganisation (ooc) and formal reorganisation (formal)

Approximately 90% of respondents rate the legal certainty of formal reorganisation as very high or high (Fig. 5). This is significantly higher (paired Wilcoxon Test, *p* value one-sided = 0.000, H3 confirmed, see Table 4) than in the case of out-of-court reorganisation (approximately 27%). This is probably due to the judicial administration in the formal proceedings and the detailed underlying legal provisions. The comparatively low level of agreement on legal certainty in connection with out-of-court reorganisation is likely related to the risks of legal challenge, especially if the restructuring efforts fail.

**Table 4:** Results for legal certainty (H3) for out-of-court reorganisation (ooc) and formal reorganisation (formal)

Test	*p* value		Statistics (406 valid cases)		Decision
Paired Wilcoxon Test	One-sided	0.000	# formal higher	324	H3 confirmed
			# ooc equal formal	71	
			# formal lower	11	
Correlation	Two-sided	0.256	Kendall-Tau-b	0.050	No correlation
		0.254	Spearman-Rho	0.057	

For legal certainty, correlations between the assessments are not significant (Table 4).

**Fig. 6:**
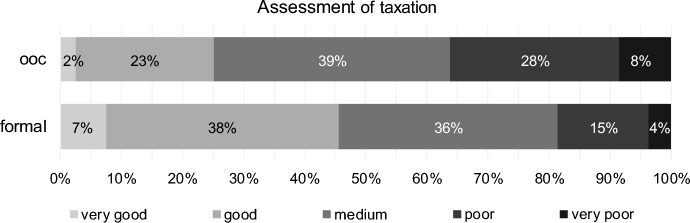
Taxation for out-of-court reorganisation (ooc) and formal reorganisation (formal)

With regard to taxation, approximately 45% of respondents rated it as very good or good in the case of formal reorganisation (Fig. 6), which is significantly better (paired Wilcoxon Test, *p* value one-sided = 0.000, H4 confirmed, see Table 5) than for out-of-court reorganisation (approximately 25%, see Fig. 6). This is obviously a deficit of out-of-court reorganisations. These results should be viewed particularly in relation to the reorganisation privilege prevailing in Austria, according to which reorganisation profits from formal proceedings are only taxed on a quota basis.

**Table 5:** Results for taxation (H4) for out-of-court reorganisation (ooc) and formal reorganisation (formal)

Test	*p* value		Statistics (401 valid cases)		Decision
Paired Wilcoxon Test	One-sided	0.000	# formal better	143	H4 confirmed
			# ooc equal formal	221	
			# formal worse	37	
Correlation	Two-sided	0.000	Kendall-Tau-b	0.224	Concordant correlation
		0.000	Spearman-Rho	0.243	

Ordinal correlation coefficients indicate that turnaround professionals who assess taxation as (very) good for ooc also assess it as good for formal (concordant correlation), significant using Kendall-Tau-b as well as Spearman-Rho. Therefore, turnaround professionals assess taxation of the two types of reorganisation similarly but slightly better for formal reorganisation. The result is significant, but this has to be interpreted carefully because of the high number of ties in the assessments (221 cases with ooc equal formal, see Table 5).

No significant differences for assessment of taxation are found concerning profession of respondents, clients’ size, own firm size or expert status.

### Process and handling

**Fig. 7:**
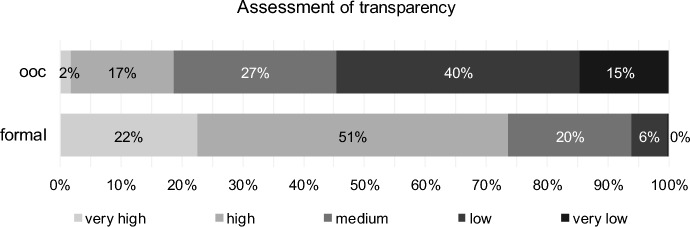
Transparency for out-of-court reorganisation (ooc) and formal reorganisation (formal)

In terms of transparency during the reorganisation process, respondents assess formal reorganisation significantly (paired Wilcoxon test, *p* value one-sided = 0.000, H5 confirmed, see Table 6) higher. Approximately 73% perceive a very high or high level of transparency in formal reorganisation proceedings (Fig. 7). In comparison, only approximately 19% of respondents rate out-of-court settlement as transparent. This indicates that many stakeholders are critical of out-of-court reorganisations without court supervision.

**Table 6:** Results for transparency (H5) for out-of-court reorganisation (ooc) and formal reorganisation (formal)

Test	*p* value		Statistics (403 valid cases)		Decision
Paired Wilcoxon Test	One-sided	0.000	# formal higher	297	H5 confirmed
			# ooc equal formal	89	
			# formal lower	17	
Correlation	Two-sided	0.967	Kendall-Tau-b	0.002	No correlation
		0.953	Spearman-Rho	0.003

Correlations between the assessments are not significant (Table 6).

**Fig. 8:**
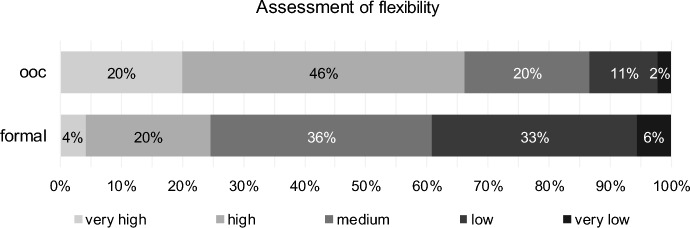
Flexibility for out-of-court reorganisation (ooc) and formal reorganisation (form)

Conversely, approximately 66% of respondents rate flexibility in out-of-court reorganisation as very high or high. In contrast, more than 39% rate flexibility in formal reorganisation as low or very low (Fig. 8).

Ordinal correlation coefficients indicate that turnaround professionals who assess flexibility as high for out-of-court reorganisation assess it as low for formal reorganisation (discordant correlation), significant using Kendall-Tau-b as well as using Spearman-Rho (Table 7).

**Table 7:** Results for flexibility (H6) for out-of-court reorganisation (ooc) and formal reorganisation (form)

Test	*p* value		Statistics (404 valid cases)		Decision
Paired Wilcoxon Test	One-sided	0.000	# ooc higher	260	H6 confirmed
			# ooc equal formal	77	
			# ooc lower	67	
Correlation	Two-sided	0.000	Kendall-Tau-b	− 0.187	Discordant correlation
		0.000	Spearman-Rho	− 0.214	

**Fig. 9:**
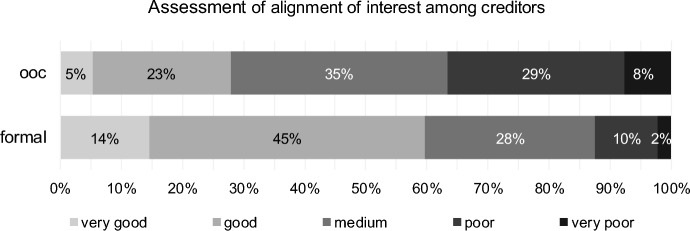
Alignment of interest among creditors for out-of-court reorganisation (ooc) and formal reorganisation (formal)

In terms of the alignment of interest among creditors during the reorganisation process, respondents assess formal reorganisation significantly (paired Wilcoxon test, *p* value one-sided = 0.000, H7 confirmed, see Table 8) higher. Approximately 59% perceive a very good or good alignment of interest among creditors in formal reorganisation proceedings (Fig. 9). In comparison, 28% of respondents rate out-of-court alignment as (very) good.

**Table 8:** Results for alignment of interest among creditors (H7) for out-of-court reorganisation (ooc) and formal reorganisation (formal)

Test	*p* value		Statistics (406 valid cases)		Decision
Paired Wilcoxon Test	One-sided	0.000	# formal better	230	H7 confirmed
			# ooc equal formal	97	
			# formal worse	79	
Correlation	Two-sided	0.000	Kendall-Tau-b	− 0.147	Discordant correlation
		0.001	Spearman-Rho	− 0.169

Ordinal correlation coefficients indicate that turnaround professionals who assess alignment of interest among creditors as good for out-of-court reorganisation assess it as poor for formal reorganisation (discordant correlation). Ordinal correlation is significant using Kendall-Tau-b as well as Spearman-Rho (Table 8).

The assessments of transparency (Kendall-Tau-b = − 0.237), flexibility (Kendall-Tau-b = − 0.223) and alignment of interest for out-of-court reorganisation (Kendall-Tau-b = − 0.228) are significantly influenced by clients’ size (all *p* values < 0.001). The bigger the clients’ size, the higher the assessment of transparency and flexibility, and the better the assessment of alignment of interest among creditors, respectively.

**Fig. 10:**
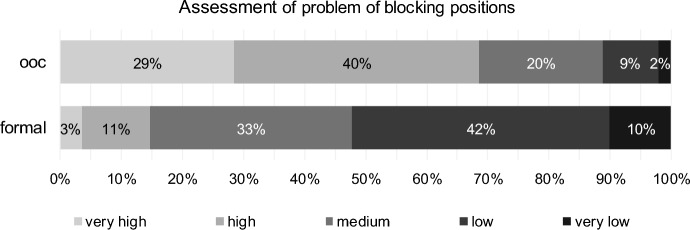
Problem of blocking positions for out-of-court reorganisation (ooc) and formal reorganisation (formal)

The problem of blocking positions, i.e., individual creditors who do not agree to a reorganisation agreement for specific reasons, is predominantly present in out-of-court reorganisation. Approximately 69% of respondents rated these problems as high or very high (Fig. 10). Only approximately 14% of respondents identify this problem in formal reorganisation (high or very high approval), which is significantly lower than in out-of-court reorganisation (paired Wilcoxon test, *p* value one-sided = 0.000, H8 confirmed, see Table 9).

**Table 9:** Results for problem of blocking positions (H8) for out-of-court reorganisation (ooc) and formal reorganisation (formal)

Test	*p* value		Statistics (407 valid cases)		Decision
Paired Wilcoxon Test	One-sided	0.000	# ooc higher	278	H8 confirmed
			# ooc equal form	89	
			# ooc lower	40	
Correlation	Two-sided	0.000	Kendall-Tau-b	− 0.193	Discordant correlation
		0.000	Spearman-Rho	− 0.217	

Turnaround professionals who assess the problem of blocking positions high for out-of-court reorganisation assess it as low for formal reorganisation (discordant correlation). This result is significant using Kendall-Tau-b as well as using Spearman-Rho (Table 9).

### Implementation

All studied factors of implementation (financial measures, operational measures and sustainable reorganisation) differ significantly between formal reorganisation and out-of-court reorganisation.

**Fig. 11:**
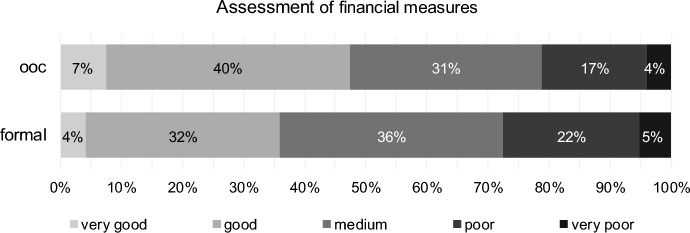
Implementation of financial measures for out-of-court reorganisation (ooc) and formal reorganisation (formal)

Approximately 47% of respondents rate financial measures as very good or good in the course of out-of-court reorganisation, and approximately 36% rate financial measures as very good or good in the course of formal reorganisation (Fig. 11).

As Hypotheses H9 to H11 are postulated to be two-sided, Tables 10, 11 and 12 show the two-sided *p* values of the paired Wilcoxon test, indicating significant results for all hypotheses.

**Table 10:** Results for implementation of financial measures (H9) for out-of-court reorganisation (ooc) and formal reorganisation (formal)

Test	*p* value		Statistics (405 valid cases)		Decision
Paired Wilcoxon Test	Two-sided	0.002	# ooc better	155	H9 confirmed
			# ooc equal form	137	
			# ooc worse	113	
Correlation	Two-sided	0.001	Kendall-Tau-b	− 0.142	Discordant correlation
		0.001	Spearman-Rho	− 0.160

**Table 11:** Results for implementation of operational measures (H10) for out-of-court reorganisation (ooc) and formal reorganisation (formal)

Test	*p* value		Statistics (402 valid cases)		Decision
Paired Wilcoxon Test	Two-sided	0.000	# ooc better	179	H10 confirmed
			# ooc equal form	135	
			# ooc worse	88	
Correlation	Two-sided	0.005	Kendall-Tau-b	− 0.119	Discordant correlation
		0.006	Spearman-Rho	− 0.136

**Table 12:** Results for ensuring sustainable reorganisation (H11) for out-of-court reorganisation (ooc) and formal reorganisation (formal)

Test	*p* value		Statistics (405 valid cases)		Decision
Paired Wilcoxon Test	Two-sided	0.001	# ooc better	145	H11 confirmed
			# ooc equal form	150	
			# ooc worse	110	
Correlation	Two-sided	0.003	Kendall-Tau-b	− 0.126	Discordant correlation
		0.004	Spearman-Rho	− 0.143	

**Fig. 12:**
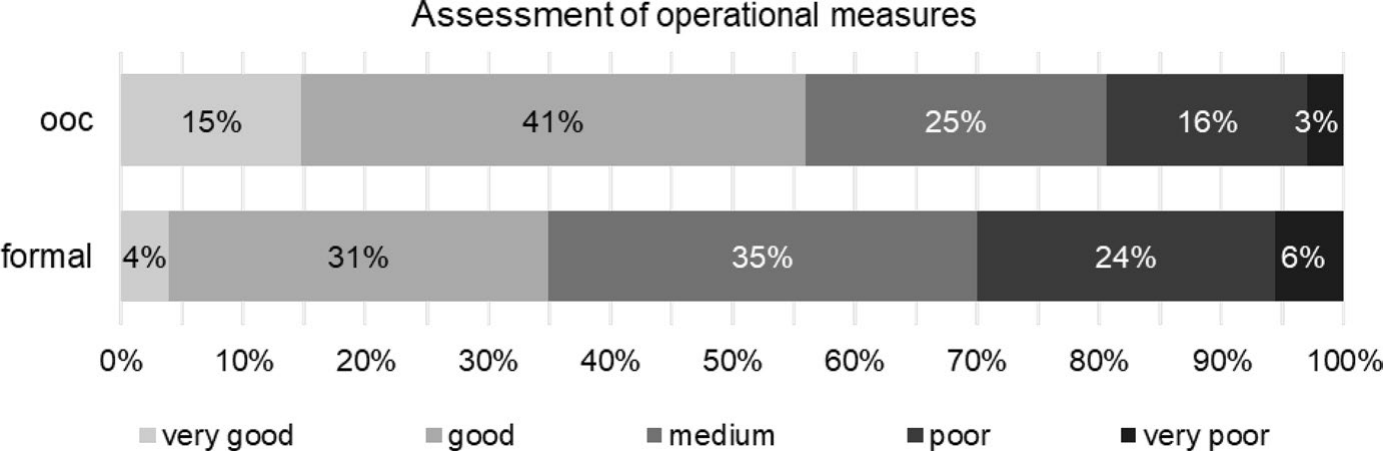
Implementation of operational measures for out-of-court reorganisation (ooc) and formal reorganisation (formal)

Approximately 56% of respondents consider the implementation of operational measures during out-of-court reorganisation to be very good or good. In contrast, 35% rate the implementation of these measures during formal proceedings as very good or good (Fig. 12).

This suggests that out-of-court reorganisation appears to be significantly more suitable for operational or performance-based reorganisation (Table 11).

**Fig. 13:**
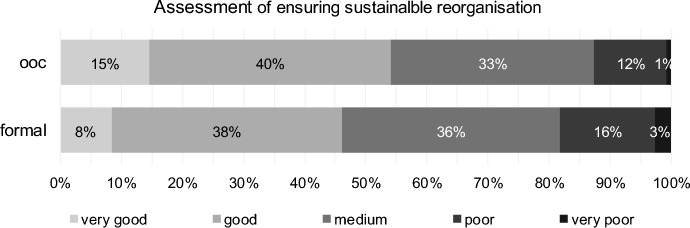
Ensuring sustainable reorganisation for out-of-court reorganisation (ooc) and formal reorganisation (formal)

There are comparatively few differences in the assessment of the success of implementing the two forms of reorganisation concerned. Based on the turnaround professionals’ assessment, both forms of reorganisation have the potential to be sustainable and therefore successful in the long term. However, out-of-court restructuring receives significantly better assessment from the professionals (Fig. 13 and Table 12).

Assessment of financial measures for out-of-court reorganisation correlates with clients’ size (Kendall-Tau-b = − 0.227, *p* value < 0.001), with expert status (Kendall-Tau-b = − 0.163, *p* value < 0.001) and, to a lower extent, with profession tax consultants (Kendall-Tau-b = 0.099, *p* value < 0.031). Assessment of operational measures for out-of-court reorganisation similarly correlates with clients’ size (Kendall-Tau-b = − 0.283, *p* value < 0.001), with expert status (Kendall-Tau-b = -0.152, *p* value < 0.001) and with profession tax consultants (Kendall-Tau-b = 0.130, *p* value < 0.005). The bigger clients’ size, the better the assessment of financial and operational measures. Tax consultants assess the measures worse than other professions; experts assess them better than non-experts.

Concerning ensuring sustainable reorganisation, the most interesting point is that clients’ size correlates negatively with out-of-court reorganisation (Kendall-Tau-b = − 0.250, *p* value < 0.001), but correlates positively with formal reorganisation (Kendall-Tau-b = 0.143, *p* value = 0.001). The bigger clients’ size, the better the assessment of ensuring sustainable reorganisation for out-of-court reorganisation, and the worse the assessment of ensuring sustainable reorganisation for formal reorganisation, respectively.

### Multivariate analysis

**Fig. 14:**
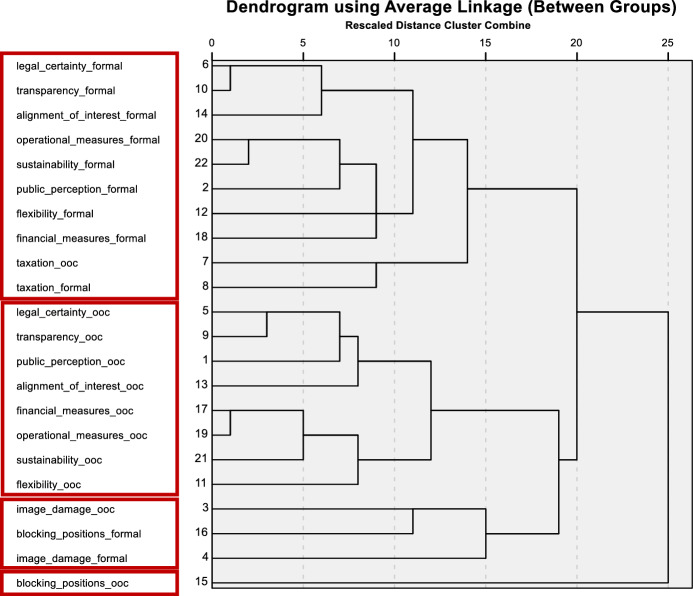
Dendogram of all assessments for out-of-court reorganisation (ooc) and formal reorganisation (formal)

Hierarchical agglomerative clustering was used to identify homogenous groups of criteria. Each of 22 criteria (11 once for formal reorganisation and once for ooc) starts in its own cluster. The result of the bottom-up approach can be seen in the dendrogram (Fig. 14), which leads to four distinct clusters.

The first cluster—the “formal cluster”—contains nearly all criteria concerning formal reorganisation as well as the assessment of taxation in ooc reorganisation, probably due to severe differences between ooc reorganisation and formal reorganisation concerning taxation. This first cluster does not include image damage as well as blocking positions for formal reorganisation. According to the results, taxation plays a special role in the assessment and probably represents one of the central advantages of formal reorganisation in the current legal situation in Austria.

The second cluster—the “ooc cluster”—contains nearly all criteria concerning ooc reorganisation, with the following exceptions: Image damage, blocking positions and taxation for ooc reorganisation are included in other clusters. The central implication of this second cluster is that a large proportion of the characteristics of out-of-court reorganisation are assessed very homogeneously by the respondents.

The remaining two clusters are much smaller: Cluster three—the “image damage cluster”—contains both criteria of image damage as well as blocking positions for formal reorganisation. This cluster contains two very extreme positions: the first is image damage, which is a significant disadvantage for formal reorganisation; the second is the problem of blocking positions, which is a significant advantage of formal reorganisation. The fourth and final cluster—“blocking positions in ooc cluster”—is a single criterion cluster for blocking positions in ooc reorganisation, which underscores the problem of blocking positions as a key challenge to the success of out-of-court reorganisations. Consequently, image damage is considered a major problem within formal reorganisation, possibly resulting from stigmatisation. This can make entrepreneurial restart more difficult. The handling of blocking positions of single creditors represents another very specific issue for reorganisations. Holdout problems are mainly prevalent in ooc reorganisation and may hinder reorganisation.

## Concluding remarks

### Discussion

Functioning insolvency law or reorganisation options for failing companies are key success factors for countries and economies in general; more specifically, they contribute to the economic recovery of viable companies. Consequently, bankruptcy and reorganisation represent a pertinent research field comprising many distinct strands (Staszkiewicz & Morawska, 2019). Substantial research has been conducted on the different forms and ways of resolving financial distress. From a legal point of view, different types of bankruptcy law have been developed and analysed concerning reorganisation options, costs or efficiency. Our analysis is informed by a neoinstitutional view of reorganisation: Bankruptcy law and company law provide the institutional framework and influence the decision of an entrepreneur to initiate reorganisation and the decision on what specific form of reorganisation (formal bankruptcy proceedings versus out-of-court settlement). Our literature review allowed us to elaborate several advantages and disadvantages of the two reorganisation forms. We clustered the results into distinctive criteria and possible influencing factors for each specific form of reorganisation: constitutions and institutional settings, processing and handling, and implementation of a reorganisation. These criteria vary across jurisdictions and cultural regions.

Internationally, especially in Europe, observers have noted trends at the legislative level towards facilitating the possibilities for reorganisation within the framework of formal proceedings and towards improving out-of-court reorganisation possibilities (European Commission, 2019; Garrido, 2012; European Commission, 2011a). Austrian bankruptcy law is traditionally regarded as a creditor-oriented system. According to Blazy et al. (2008), it belongs to the prosecured creditor model, which protects the interests of secured creditors. Although the idea of reorganisation is deeply rooted in Austrian bankruptcy law, out-of-court reorganisation is also of great practical importance, with comparably high rates of success (Mayr et al., 2020; Mayr, 2018; European Commission, 2011b). Our findings confirm the results of García-Posada Goméz et al. (2018), according to which there is a substantial need for legal options for out-of-court restructuring. With the implementation of the directive of the European Commission by the Austrian Restructuring Regulation (“Restrukturierungsordnung”, entry into force on July 17, 2021), the framework conditions and possibilities for out-of-court reorganisation have already been significantly expanded. The new procedure is positioned between traditional court-supervised procedures and out-of-court reorganisation that were already being used.

Since many detailed aspects of both a legal and a business nature must be considered when deciding on a particular form of reorganisation, managers and entrepreneurs often call on the expertise of turnaround professionals (Decker, 2018; Kanter, 2003). In our study, we therefore investigated how turnaround professionals valuate the distinctive criteria and influencing factors for a certain form of reorganisation in a creditor-friendly bankruptcy system, such as the Austrian system. With the help of a survey of turnaround professionals in Austria, our empirical study addresses the question of how turnaround professionals evaluate the decision criteria for a specific form of reorganisation in a creditor-friendly bankruptcy system.

The results of the survey indicate that both formal and out-of-court reorganisation offer specific advantages, since all relevant variables differ significantly between formal proceedings and out-of-court reorganisation. The choice of the appropriate form of reorganisation must therefore be made on a case-by-case basis, depending on the overall circumstances. Our study shows significant differences with regard to the assessment of constitutions and institutional settings and may serve as a guide in the decision-making process for those involved in a reorganisation. A general problem with restructuring is the possible damage to the company's image that can result from stigmatisation within the company's environment. According to the turnaround experts surveyed, the public perception of out-of-court restructuring is much better than that of formal restructuring. The potential image damage to a company is estimated to be much higher in formal reorganisation, most likely because of the public announcement of every insolvency required by Austrian law. This is partly due to the fact that a large proportion of out-of-court reorganisations take place discreetly and without publicity (Fischer & Wahrenburg, 2012; Mayr, 2018).

However, formal proceedings offer advantages in taxation and legal certainty, as deficits and disadvantages have, in the past, emerged within Austria’s legal provisions for out-of-court reorganisations. Regarding the tax advantage of formal reorganisation in Austria, it should be noted that this can lead to a considerable advantage in the liquidity of the company compared to out-of-court reorganisations. This makes it easier to plan and finance the reorganisation (Blazy et al., 2008). The very positive assessment of the legal certainty of formal reorganisation likely indicates confidence in the courts but also suggests efficient handling of the proceedings. Regarding the set of criteria subsumed under process and handling of the reorganisation, out-of-court reorganisation is perceived as more flexible in handling. This is particularly important with regard to deadlines, individual agreements with individual creditors and quotas to be paid. Transparency is, in essence, an argument for formal proceedings. As the analysis of the results shows, the criteria of flexibility and transparency cannot be seen in isolation; rather, they are opposed to each other in the procedural paths. The decision for a specific form of reorganisation has to be made in weighting the importance of these criteria, from the point of view of both the entrepreneurs and the creditors. For formal proceedings, our results show a significantly lesser problem in the handling of blocking positions. This circumstance has already been considered in the implementation of the Austrian restructuring regulation (“Restrukturierungsordnung”) by means of a “cram-down”. Consequently, the handling of the blocking positions of single creditors represents a very specific issue for reorganisations. Holdout problems are mainly prevalent in out-of-court reorganisation and may hinder reorganisation.

Regarding the implementation of a reorganisation, a significant proportion of the experts surveyed consider both forms to have at least a good possibility of implementation for all criteria (financial and operational measures, sustainable reorganisation). In practice, only the elimination of the causes of the crisis and an appropriate mix of financial and operational measures will lead to sustainable recovery (Mayr et al., 2017). Consequently, our findings indicate that, in practice, there is a need for both reorganisation forms. When deciding on a form of reorganisation, the circumstances of the individual case must be examined. From a practical point of view, the fact that it is easier to implement performance-related or operational restructuring measures seems to be a potential argument in favour of out-of-court reorganisation. If, on the other hand, a reorganisation is exhibiting signs of potential failure due to problems in handling blocking positions, or if the highest possible degree of transparency is required in the course of the liquidation, formal proceedings might be warranted.

### Limitations and further research

The representativeness of the sample used in our study cannot be guaranteed due to a lack of information on the total population and a relatively low response rate (possibly indicating a self-selection bias). All quantitative results should be regarded as exploratory but nonetheless meaningful, inasmuch as a low response rate does not necessarily make results uninformative (Meterko et al., 2015). Moreover, to verify the quality of the data, we controlled for non-response bias, comparing the first third of the respondents with the last third of the respondents. There was no indication of non-response bias.

We acknowledge that our results must be interpreted against the background of Austria’s prosecured creditor insolvency law, including a very significant tax advantage for formal reorganisation. A second limitation relates to the fact that the survey was carried out before implementation of the Austrian restructuring regulation (“Restrukturierungsordnung”). However, since only little empirical data is available for Austria to date, the results may provide good indications for the future development of framework conditions in both Austria and other countries.

Our study adds to the literature by clustering several distinctive criteria and influencing factors for a specific form of reorganisation (Blazy et al., 2008, 2014; Fischer & Wahrenburg, 2012; Gilson, 1989; Jacobs et al., 2012; Kim, 2006). These criteria can be used for further research in the field of bankruptcy law and reorganisation. Furthermore, we have identified a range of distinct criteria that favour one form or the other. The identified criteria can thus be used for further research as well as for the further practical development of reorganisation options. Practitioners such as entrepreneurs or consultants can provide a starting point for concrete reorganisation decisions. In line with the Commission's call for a preventive, out-of-court, restructuring framework applicable at the Union level—intended to enable debtors to restructure to avoid insolvency and ensure the viability of their companies (European Commission, 2019) –, our results provide evidence for improvement. On the one hand, a key starting point for improving the framework conditions for formal reorganisation is improving the public perception of bankruptcy and subsequent reorganisation. Information campaigns can bring about change in the medium term (European Commission, 2011b). On the other hand, legislators must be aware that out-of-court reorganisation has very significant disadvantages, including the problem of blocking positions as well as taxation. Such disadvantages must also be considered in the further development of preventive restructuring frameworks in the member states.

Future research may also address the trade-off between competing aspects (e.g., flexibility versus transparency). A further need for research can be recognised in the direction of improving public perception and reducing image damage in connection with formal proceedings.

## Footnotes

1 In this paper, the stated terms will be used as synonyms. In the context of banks, the term "workouts" is frequently used. Accordingly, so-called workout departments in banks have the task to professionally process and support credit customers with an increased risk of default (Gertner and Scharfstein 1991).

2 For a detailed overview of Austrian insolvency law, see also Konecny and Reisch (2014).

3 All postulated research hypotheses are alternative hypotheses in statistical inference.

## References

[CR1] Armstrong JS, Overton TS (1977). Estimating nonresponse bias in mail surveys. Journal of Marketing Research.

[CR2] Baird, S. R. (2014). An empirical investigation of successful, high performing turnaround professionals: Application of the dynamic capabilities theory. *Dissertation*, 1–188.

[CR3] Blazy R, Chopard B, Fimayer A (2008). Bankruptcy law: A mechanism of governance for financially distressed firms. European Journal of Law and Economics.

[CR4] Blazy R, Martel J, Nigam N (2014). The choice between informal and formal restructuring: The case of French banks facing distressed SMEs. Journal of Banking & Finance.

[CR5] Blazy R, Stef N (2020). Bankruptcy procedures in the post-transition economies. European Journal of Law and Economics.

[CR6] Bosse DA, Phillips RA, Harrison JS (2009). Stakeholders, reciprocity, and firm performance. Strategic Management Journal.

[CR7] Brownstein HB, Morris TW (2002). The truth about turnaround professionals. CPA Journal.

[CR8] Camacho-Miñano M-M, Pascual-Ezama D, Urquía-Grande E (2013). On the efficiency of bankruptcy law: Empirical evidence in Spain. International Insolvency Review.

[CR80] Cardon MS, Stevens CE, Potter DR (2011). Misfortunes or mistakes?. Journal of Business Venturing.

[CR9] Chowdhury F, Terjesen S, Audretsch D (2015). Varieties of entrepreneurship: Institutional drivers across entrepreneurial activity and country. European Journal of Law and Economics.

[CR10] Coase RH (1937). The Nature of the Firm. Economica.

[CR11] Commons JR (1950). The economics of collective action.

[CR12] Conklin BC, Michelin AM (2004). White knight wanted: What to expect from restructuring consultants. Journal of Business Strategy.

[CR13] Damaraju NL, Barney JB, Dess GG (2020). Do stringent bankruptcy laws always deter entrepreneurial activities? A study of cultural influences. Entrepreneurship Theory and Practice.

[CR14] Davydenko SA, Franks JR (2008). Do bankruptcy codes matter? A study of defaults in France, Germany, and the U.K. The Journal of Finance.

[CR15] Decker C (2018). Stakeholders’ impact on turnaround performance: The case of German savings banks. Journal of Small Business Management.

[CR16] DiMaggio PJ, Powell WW (1983). The iron cage revisited: Institutional isomorphism and collective rationality in organizational fields. American Sociological Review.

[CR17] DiMaggio PJ, Powell WW, Powell WW, DiMaggio PJ (1991). Introduction. The New institutionalism in organizational analysis.

[CR18] Dugger WM (1979). Methodological differences between institutional and neoclassical economics. Journal of Economic Issues.

[CR19] European Commission. (2011a). A second chance for entrepreneurs: Prevention of bankruptcy, simplification of bankruptcy procedures and support for a fresh start: European Commission Brussels.

[CR20] European Commission. (2011b). Business Dynamics: Start-ups, Business Transfers and Bankruptcy. European Commission Brussels.

[CR21] European Commission. (2019). Directive (EU) 2019/1023 of the European Parliament and of the Council of 20 June 2019 on preventive restructuring frameworks, on discharge of debt and disqualifications, and on measures to increase the efficiency of procedures concerning restructuring, insolvency and discharge of debt, and amending Directive (EU) 2017/1132 (Directive on restructuring and insolvency).

[CR22] Fischer M, Wahrenburg M (2012). The resolution of corporate distress inside and outside of the courtroom. Working Paper.

[CR23] Franks JR, Torous WN (1989). An empirical investigation of U.S. firms in reorganization. The Journal of Finance.

[CR24] Frouté P (2007). Theoretical foundation for a debtor friendly bankruptcy law in favour of creditors. European Journal of Law and Economics.

[CR25] García-Posada Gómez M, Vegas Sánchez R (2018). Bankruptcy reforms in the midst of the Great Recession: The Spanish experience. International Review of Law and Economics.

[CR26] Garrido, J. M. (2012). Out-of-court debt restructuring. Washington: World Bank (World Bank studies).

[CR27] Gertner R, Scharfstein D (1991). A theory of workouts and the effects of reorganization law. The Journal of Finance.

[CR28] Gilson S (1989). Management turnover and financial distress. Journal of Financial Economics.

[CR29] Gilson S (1991). Managing default: some evidence on how firms choose between workouts and chapter 11. Journal of Applied Corporate Finance.

[CR30] Hotchkiss, E. S., John, K., Mooradian, R. M., & Thorburn, K. S. (2008). Bankruptcy and the resolution of financial distress. In B. E. Eckbo (Ed.), *Handbook of empirical corporate finance* (pp. 235–287). North Holland. 10.1016/B978-0-444-53265-7.50006-8

[CR31] Hotchkiss, E. S., Strömberg, P. J., & Smith, D. C. (2014). Private Equity and the Resolution of Financial Distress. AFA 2012 Chicago Meetings Paper; *ECGI - Finance Working Paper* No. 331/2012, 1–58. 10.1093/rcfs/cfab015

[CR32] Jacobs M, Karagozoglu AK, Layish DN (2012). Resolution of corporate financial distress: An empirical analysis of processes and outcomes. The Journal of Portfolio Management.

[CR33] James SD (2016). Strategic bankruptcy: A stakeholder management perspective. Journal of Business Research.

[CR34] Kaiser K (1996). European bankruptcy laws: Implications for corporations facing financial distress. Financial Management.

[CR35] Kanter, R. M. (2003). Leadership and the psychology of turnarounds. *Havard Business Review,* 58–67. 12800717

[CR36] Kim D-K (2006). Do self-serving managers choose chapter 11 filing over out-of-court restructuring?. Journal of Economics and Finance.

[CR37] Kim D-K, Kwok CCY (2009). The influence of managerial incentives on the resolution of financial distress. Review of Quantitative Finance and Accounting.

[CR38] Konecny, A., & Reisch, U. (2014). Österreichische Insolvenzordnung/Austrian Insolvency Act (1. edition). NWV Verlag.

[CR39] Laryea, T. (2010). Approaches to Corporate Debt Restructuring in the Wake of Financial Crisis. IMF Staff Position Note.

[CR40] Lee S-H, Yamakawa Y (2012). Forgiving features for failed entrepreneurs vs. cost of financing in bankruptcies. Management International Review.

[CR41] Lee S-H, Peng MW, Barney JB (2007). Bankruptcy law and entrepreneurship development: A real options perspective. Academy of Management Review.

[CR42] Lee S-H, Yamakawa Y, Peng MW, Barney JB (2011). How do bankruptcy laws affect entrepreneurship development around the world?. Journal of Business Venturing.

[CR43] Leslie LL (1972). Are high response rates essential to valid surveys?. Social Science Research.

[CR44] LoPucki LM, Doherty JW (2008). Professional overcharging in large bankruptcy reorganization cases. Journal of Empirical Legal Studies.

[CR45] Manzaneque M, Merino E, Sánchez JA (2021). Survival of financially distressed SMEs and out-of-court versus in-court reorganization: Explanatory internal factors. Spanish Accounting Review.

[CR46] Mayr S, Mitter C, Aichmayr A (2017). Corporate crisis and sustainable reorganization: Evidence from bankrupt Austrian SMEs. Journal of Small Business Management.

[CR47] Mayr S (2018). Die außergerichtliche Unternehmenssanierung aus betriebswirtschaftlicher Sicht.

[CR48] Mayr S, Duller C, Stumbauer K (2020). House banks in out-of-court reorganization: Evidence from Austria. The Journal of Entrepreneurial Finance.

[CR49] McCarthy PT, O'Riordan C, Griffin R (2014). The other end of entrepreneurship: A narrative study of insolvency practice in Ireland. International Journal of Entrepreneurial Behavior & Research.

[CR50] Meterko M, Restuccia JD, Stolzmann K, Mohr D, Brennan C, Glasgow J, Kaboli P (2015). Response rates, nonresponse bias, and data quality: Results from a national survey of senior healthcare leaders. Public Opinion Quarterly.

[CR51] Mitter C (2015). Bankruptcy law as a corporate governance mechanism: Issues and evidence. European Journal of Management.

[CR52] Mruk E, Aguiar-Díaz I, Ruiz-Mallorquí MV (2019). Use of formal insolvency procedure and judicial efficiency in Spain. European Journal of Law and Economics.

[CR53] Mutanen, J.‑P., & Lehtimäki, M. J. (2009). Favourable for private workouts. *International Financial Law Review (Financial Crisis Supplement)*, 21–24.

[CR54] North DC (1990). Institutions, institutional change, and economic performance.

[CR55] Nikolaou I, Gouras A, Vakola M, Bourantas D (2007). Selecting change agents: Exploring traits and skills in a simulated environment. Journal of Change Management.

[CR56] Peng MW, Yamakawa Y, Lee S-H (2010). Bankruptcy laws and entrepreneur-friendliness. Entrepreneurship Theory and Practice.

[CR57] Phelan JC, Link BG, Dovidio JF (2008). Stigma and prejudice: One animal or two?. Social Science & Medicine.

[CR58] Sautner Z, Vladimirov V (2018). Indirect costs of financial distress and bankruptcy law: Evidence from trade credit and sales*. Review of Finance.

[CR59] Semadeni M, Cannella AA, Fraser DR, Lee DS (2008). Fight or flight: Managing stigma in executive careers. Strategic Management Journal.

[CR60] Simmons SA, Wiklund J, Levie J (2014). Stigma and business failure: Implications for entrepreneurs’ career choices. Small Business Economics.

[CR61] Singh S, Corner PD, Pavlovich K (2015). Failed, not finished: A narrative approach to understanding venture failure stigmatization. Journal of Business Venturing.

[CR62] Staszkiewicz P, Morawska S (2019). The efficiency of bankruptcy law: Evidence of creditor protection in Poland. European Journal of Law and Economics.

[CR63] Stef N, Bissieux J-J (2022). Resolution of corporate insolvency during COVID-19 pandemic. Evidence from France, International Review of Law and Economics.

[CR64] Sudarsanam S, Lai J (2001). Corporate financial distress and turnaround strategies: An empirical analysis. British Journal of Management.

[CR65] Voigt S (2016). Determinants of judicial efficiency: A survey. European Journal of Law and Economics.

[CR66] Waisman, S. Y., & Lucas, J. W. (2008). The role and retention of the chief restructuring officer. *The Americas Restructuring and Insolvency Guide*, 200–205.

[CR67] Wiesenfeld BM, Wurthmann KA, Hambrick DC (2008). The stigmatization and devaluation of elites associated with corporate failures: A process model. Academy of Management Review.

[CR68] Williamson OE (2000). The new institutional economics: Taking stock, looking ahead. Journal of Economic Literature.

[CR69] Yost, K. E. (2002). The choice among traditional Chapter 11, prepackaged bankruptcy, and out -of -court restructuring. *Dissertation*.

[CR70] Zafiris N (2018). Why we should change our attitude towards distressed firms. Economic Affairs.

[CR71] Zucker LG (1987). Institutional theories of organization. Annual Review of Sociology.

